# Spindle cell lipoma of the larynx: A case report

**DOI:** 10.1097/MD.0000000000021118

**Published:** 2020-07-17

**Authors:** Wang Qin-ying, Zhou Shui-hong, Liu Yong-cai, Chen Hai-hong

**Affiliations:** Department of Otolaryngology, First Affiliated Hospital, College of Medicine, Zhe Jiang University, Hangzhou, PR China.

**Keywords:** coblation, larynx, spindle cell lipoma

## Abstract

**Introduction:**

Lipomas are rarely found in primary mesenchymal tumors of the hypopharynx and larynx. When they do appear, they often macroscopically resemble a retention cyst or laryngeal nodule. The laryngeal spindle cell lipoma, a variant, is extremely rare.

**Patient concerns:**

A 65-year-old woman presented with a 3-month history of pharyngeal paraesthesia. Laryngoscopy revealed the presence of a well-encapsulated, smooth-surfaced, yellowish, pedicled mass on the left epiglottis. Magnetic resonance imaging confirmed the epiglottic mass.

**Diagnosis:**

Following excision of the mass, the diagnosis of an spindle cell lipoma was established and confirmed by immunohistochemistry.

**Interventions:**

Surgical excision of the lesion using a controlled-temperature plasma technique (coblation).

**Outcomes:**

At the last (4-year) follow-up evaluation, the patient was asymptomatic and without recurrence.

**Conclusion:**

SLC involvement of the epiglottis is rare. Coblation is an effective means to remove it.

## 1 Introduction

Lipomas are the most frequently observed soft tissue tumors in adults. One subtype is the spindle cell lipoma (SCL), which typically presents as a benign lipomatous neoplasm in the posterior neck and back of older men. It accounts for approximately 1.5% of all lipomas.^[1]^ The most common location for classic oral and maxillofacial lipomas is the parotid region, followed by the buccal mucosa, although previous reports have documented SCLs arising in such rare sites as the face, forehead, upper arm, thigh, wrist, and hand.^[2–4]^ It may rarely be seen in the epiglottis. We report our experience with a 65-year-old woman who presented with pharyngeal paraesthesia.

## 2 Case report

This study was approved by the Ethics Committee of the First Affiliated Hospital, College of Medicine, Zhe Jiang University, China (approval no. 2015056). Written informed consent was obtained from the patient for publication of this case report and the accompanying images.

**Figure 1:**
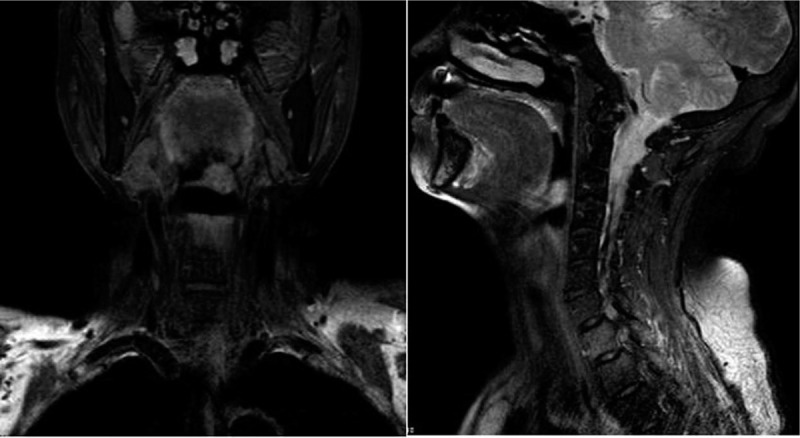
Magnetic resonance imaging shows a non-homogeneous soft tissue mass arising from the left epiglottis.

A 65-year-old woman was admitted to our hospital with a 3-month history of pharyngeal paraesthesia. She did not complain of, or exhibit, hoarseness, choking spells, stridor, or dyspnea. She was a non-smoker. Laryngoscopy revealed the presence of a well-encapsulated, smooth-surfaced, yellowish, pedicled mass arising from the left epiglottis. Magnetic resonance imaging subsequently confirmed the presence of the non-homogeneous soft tissue mass (Fig. 1). There was no evidence of enlarged cervical lymph nodes. With the patient under general anesthesia, epiglottectomy was performed via microlaryngoscopy using a temperature-controlled plasma technique (coblation). The patient's pharyngeal paraesthesia resolved after removing the mass. The patient's voice is currently normal, although she choked when drinking water after the surgery. She underwent rehabilitation for 5 days, which was successful.

**Figure 2:**
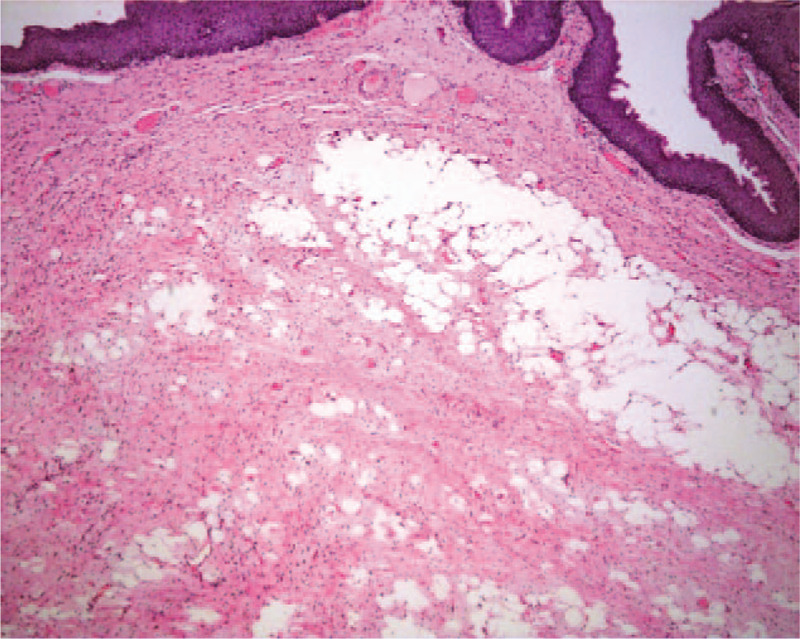
Histologically, the tumor was composed of mature adipocytes and proliferation of less atypical spindle cells on a ropey collagen background. × 40.

**Figure 3:**
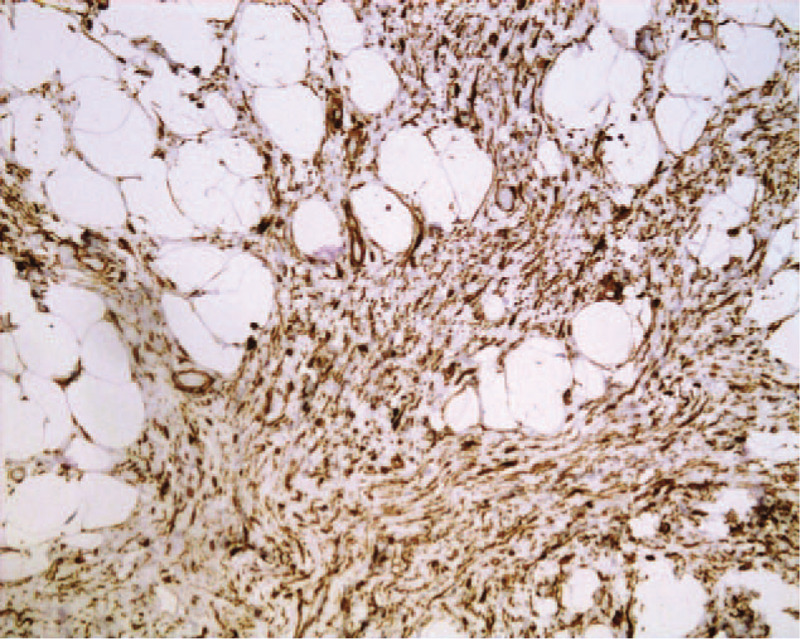
Tumor cells were diffusely positive for CD34.

Histologic examination of the specimen showed proliferation of spindle cells with elongated cytoplasmic processes on a loose, edematous, myxoid background (Fig. 2). Immunohistochemical staining showed spindle cells that were diffusely positive for CD34 with abundant fibrous and myxoid stroma interspersed with mature fatty tissue (Fig. 3). The final diagnosis was an SCL.

The patient's course during the follow-up period was uneventful, with no evidence of disease recurrence at the 4-year postoperative follow-up evaluation.

## 3 Discussion

SCL is rare, accounting for approximately 1.5% of all lipomas.^[1]^ It has a 10:1 predilection for the male sex, with the mean age of occurrence at 56 years.^[5]^ In the present case, the patient was a 65-year-old woman. Clinically, the lingual SCL often presents as a painless, slowly enlarging mass in patients 31 to 78 years of age, with male predominance. The yellowish color and soft consistency of the lesion in our patient suggested a lipoma, although it required immunohistochemical staining for a definitive diagnosis.

Histologically, SCLs are classically composed of mature fat cells, bland spindle cells with low mitotic activity, ropey collagen, and a myxoid matrix. The differential diagnosis is important for tumors that share histological features with SCL, such as the well-differentiated sclerosing liposarcoma.^[6]^ Immunohistochemistry had revealed spindle cell positivity for CD34, which has been used as a diagnostic marker for SCL.^[7]^ In the present case, the tumor cells did indeed exhibit immunoreactivity for CD34.

In contrast to the well-differentiated liposarcoma, which occurs in various locations, SCL mainly appears in subcutaneous regions of the upper back, neck, or shoulder, with uncommon occurrences at other sites.^[2,4]^ Histopathologically, SCLs are characterized by mature fat cells admixed with spindle cells arranged in prominent collagen bundles in a lobular pattern. The proportion of spindle cells can vary. For example, some tumors are predominantly composed of mature adipocytes and a few scattered spindle cells, whereas others contain mostly spindle cells and only a small number of mature adipocytes. In the current case, the spindle cell component represented a moderate proportion of the tumor. Overall, the clinical, histopathological, and immunohistochemical profile of the present case supports the SCL diagnosis.

The preferred method of treatment for laryngeal and hypopharyngeal lipogenic tumors is radical excision of the lesion, ideally under endoscopic vision.^[8,9]^ Complete resection is possible with this approach because of the nature and location of these lesions, as shown in the current patient who underwent coblation via microlaryngoscopy. The patient was monitored during a 4-year follow-up period despite the low recurrence rates reported for SCLs, and we found no evidence of symptoms or recurrent disease. Thus, complete surgical resection with the temperature-controlled plasma technique is a useful procedure for addressing an SCL, a rare tumor involving the epiglottis.

## Acknowledgments

We thank Nancy Schatken BS, MT(ASCP), from Liwen Bianji, Edanz Group China (www.liwenbianji.cn/ac), for editing the English text of a draft of this manuscript.

## Author contributions

**Data curation:** wang qinying.

**Investigation:** wang qinying.

**Resources:** wang qinying, liu yongcai.

**Software:** chen haihong.

**Supervision:** zhou shuihong.

**Visualization:** chen haihong.

**Writing – original draft:** liu yongcai, chen haihong.

**Writing – review & editing:** zhou shuihong.
